# Supplementary material for: Impact of High-to-Moderate Penetrance Genes on Genetic Testing: Looking over Breast Cancer
Source: Genes (Basel). 2023 Jul 26;14(8):1530. doi: 10.3390/genes14081530 (PMC10454640; doi:10.3390/genes14081530)
Supplement: Supplementary file 1 [file genes-14-01530-s001.zip › genes-2493637-supplementary.pdf]

Table S1: Histology and ER, PR, and HER molecular profile correlated with each variant.

	C DNA CHANGE	HISTOLOGY	ER profile	PR profile	HER2 profile
ATM (NM_000051.3)	c.6300C>G	INVASIVE MUCINOUS	POSITIVE	POSITIVE	NEGATIVE
	c.6300C>G	INVASIVE LOBULAR	POSITIVE	POSITIVE	NEGATIVE
	EXON 29 DELETION	IDC-NST	POSITIVE	POSITIVE	NEGATIVE
	c.2638+1G>A	IDC-NST	POSITIVE	POSITIVE	NEGATIVE
CHEK2 (NM_007194.3)	c.1427C>T	IDC-NST	UNKNOWN	UNKNOWN	UNKNOWN
	c.793-1G>A	IDC-NST	POSITIVE	NEGATIVE	NEGATIVE
	c.507del	IDC-NST	POSITIVE	POSITIVE	UNKNOWN
	c.470T>C	IDC-NST	NEGATIVE	NEGATIVE	UNKNOWN
	EXON 9 DELETION	IDC-NST	NEGATIVE	NEGATIVE	POSITIVE
PALB2 (NM_024675.3)	c.355C>T	IDC-NST	NEGATIVE	NEGATIVE	NEGATIVE
	c.355C>T	IDC-NST	UNKNOWN	UNKNOWN	UNKNOWN
RAD51D (NM_002878.3)	c.803G>A	IDC-NST	NEGATIVE	NEGATIVE	NEGATIVE
	c.803G>A	IDC-NST	POSITIVE/NEGATIVE	POSITIVE/NEGATIVE	NEGATIVE/POSITIVE
	c.757C>T	DUCTAL INTRAEPITHELIAL	POSITIVE	NEGATIVE	POSITIVE
RAD51C (NM_058216.2)	c.1026+5_1026+7delGTA	INVASIVE LOBULAR	POSITIVE	POSITIVE	NEGATIVE
